# Exposure to calls before hatching affects the post-hatching behaviour of domestic chickens

**DOI:** 10.1098/rsos.240114

**Published:** 2024-08-14

**Authors:** Gabriella E. C. Gall, Megan Letherbarrow, Ariana Strandburg-Peshkin, Andrew N. Radford, Joah R. Madden

**Affiliations:** ^1^ Zukunftskolleg, University of Konstanz, Konstanz, Germany; ^2^ Centre for the Advanced Study of Collective Behaviour, University of Konstanz, Konstanz, Germany; ^3^ Department of Biology, University of Konstanz, Konstanz, Germany; ^4^ Department for the Ecology of Animal Societies, Max Planck Institute of Animal Behavior, Konstanz, Germany; ^5^ Centre for Research in Animal Behaviour (CRAB), Washington Singer Laboratories, University of Exeter, Exeter, UK; ^6^ School of Biological Sciences, University of Bristol, Bristol, UK

**Keywords:** playback experiment, *Gallus gallus domesticus*, pre-hatching soundscape, pre-hatching playback, post-hatching behaviour

## Abstract

The soundscape experienced by animals early in life can affect their behaviour later in life. For birds, sounds experienced in the egg can influence how individuals learn to respond to specific calls post-hatching. However, how early acoustic experiences affect subsequent social behaviour remains unknown. Here, we investigate how exposure to maternal ‘cluck’ calls pre-hatching affects the behaviour of domestic chickens (*Gallus gallus domesticus*) at 3–5 days and 17–21 days old. We incubated eggs and played cluck calls to half of them. After hatching, we raised chicks in small groups occupying different enclosures. At 3–5 days old, we tested chicks’ responses to three stimuli: (i) background sound, (ii) chick calls and (iii) cluck calls. We found that the pre-hatching experience of cluck calls reduced the likelihood of moving in response to all three stimuli. At 17–21 days old, some chicks explored beyond their own enclosure and ‘visited’ other groups. Chicks exposed to cluck calls before hatching were three times more likely to enter another group’s enclosure than control chicks, and this was unaffected by the chicks’ social connectedness. Our results indicate age- and context-dependent responses of chicks to pre-hatching cluck-call playbacks, with potential long-term effects on individual social behaviour.

## 1. Introduction

Developmental plasticity is common in the animal kingdom, with early-life conditions influencing physiology, morphology and behaviour later in life through altered developmental processes [1–4]. Individuals do not passively react to current conditions but actively interact with their environment. First experiences and interactions can happen very early in life, with embryos already gaining information from their environment using different sensory modalities. Information may be obtained through various means, including maternal provisioning, reflecting conditions experienced by the mother [1,2,5], chemical cues (e.g. from predators) [6] or acoustic/vibrational cues [7,8]. Sound can pass across both short and long distances, with relatively little influence of physical barriers [9–11]. In addition, acoustic information can be transmitted without altering the suitability of the prenatal environment for development [4]. In birds, sound exposure during incubation can facilitate auditory learning by chicks [12], chick development [7,13], food neophobia [14], song learning [14–17] and the ability of chicks to respond appropriately to species-specific calls [18–21], including the development of escape responses triggered by caution calls [17]. These adaptations may not be a simple case of associative learning, as some acoustic cues heard before hatching can alter later-life responses to other call types which were not heard pre-hatching. For example, wood ducklings (*Aix sponsa*) exposed to sibling hatchling calls during incubation responded more selectively to maternal calls after hatching than did non-exposed chicks [21]. Similarly, ring-necked pheasant (*Phasianus colchicus*) chicks exposed to hen incubation calls before hatching showed a stronger attraction to maternal feeding and caution calls than chicks that had not been exposed to such calls during incubation [18].

Prenatal sound exposure may provide anticipatory cues for longer term environmental conditions [4] and may affect ontogenetic pathways in two different ways. First, cues about the immediate environment might affect the transition between life stages. For example, embryos of insects, reptiles and birds synchronize hatching time based on vibrational or acoustic cues from their clutch mates, parents or approaching predators [4,8]. Second, prenatal sounds might also modify individual phenotypes to suit predicted conditions in a subsequent life stage. For instance, during later incubation, zebra finch (*Taeniopygia guttata*) parents produce ‘heat-calls’ in response to high ambient temperatures [7], which modify offspring development such that embryos exposed to these calls tend to be lighter as nestlings [7], show improved mitochondrial efficiency [22] and are more heat tolerant as adults [23]. Such modifications may be specific to particular circumstances (e.g. environmental temperature or predator threat levels) or may be more general, as evidenced by changes in stress physiology and behaviour of yellow-legged gulls (*Larus michahellis*) and Japanese quail (*Coturnix coturnix japonica*) prompted by pre-hatching acoustic environments [13,24]. While it is now evident that pre-hatching sound environments can affect the response of individuals in later life to con- or heterospecific acoustic cues or environmental conditions, less is known about how such processes might affect social behaviours which are commonly underpinned by signalling, including calls, songs and other sounds [11], though see [25] for a review.

Precocial birds offer a specifically well-suited model system to investigate questions related to the effect of pre-hatching stimulation on post-hatching behaviour, as they can be kept in a laboratory setting, embryo experiences can be easily manipulated and chicks are able to move and engage with conspecifics immediately after hatching [25]. In chickens (*Gallus gallus domesticus*), hens signal to their chicks using a range of vocalizations, including cluck calls used to induce them to follow the hen to a different location [26]. Broody hens also frequently emit clucks in the last days of incubation [27]. After hatching, chicks emit two primary vocalizations: distress calls, indicating increased stress levels [28,29], and pleasure-note calls, indicating a relaxed state [26]. Here, we explore how exposure to maternal calls during incubation affects the social exploratory behaviour of domestic chicks at 3−5 days and 17−21 days of age. At 3–5 days of age, chicks had the opportunity to get used to their new environment but were still fresh to the world, while at 17 days, the chicks were able to start using their wings, thus significantly increasing mobility. We raised two cohorts of chicks in artificial incubators and controlled the pre-hatching acoustic environment so that embryos either experienced or did not experience playbacks of maternal cluck calls (*incubation treatment*). Once chicks hatched, we reared them in groups of six individuals and observed them daily. At 3−5 days post-hatching, we conducted a playback experiment (*chick treatment*) to explore how the experience of hearing maternal calls during incubation affects the behaviour and vocalizations of chicks post-hatching, predicting chicks with incubation-call treatment to be more exploratory. When chicks reached approximately 17 days old, we opportunistically observed that they were able to perch on the enclosure walls and to hop out of their own enclosures into the enclosures of other groups. To test whether chicks that had experienced pre-hatching maternal calls were more likely to ‘visit’ other groups, we analysed these opportunistic recordings.

## 2. Methods

### 2.1. Overview

**Figure 1.**
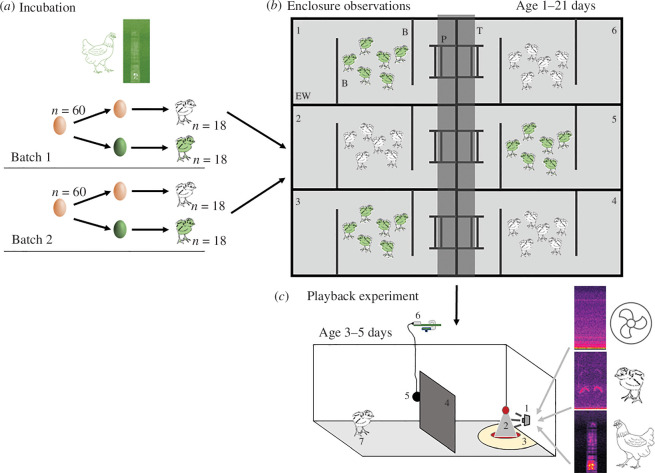
Schematic of the study and experimental protocol. (*a*) Incubation: two cohorts of eggs were incubated, with half of each cohort (green) receiving playbacks of maternal cluck calls in the final week of incubation (incubation-call treatment) and the other half (orange) receiving no playback at that stage (incubation-control treatment). (*b*) Rearing and enclosure observations: following hatching, chicks were housed in groups of six chicks each. Enclosures were arranged in two rows of three, separated by tables (T, dark grey quadrangle) in the middle. Each enclosure included a perch (P), within-enclosure barriers (B) and enclosure walls (EW) as well as ad libitum water, grit, food and a heat lamp suspended above the centre of the enclosure. The positions of enclosures containing incubation-call treatment and incubation-control treatment chicks were balanced across cohorts. In each enclosure, the behaviour of chicks was recorded daily through two Raspberry Pi cameras suspended above each enclosure. (*c*) Playback experiment with chicks when 3−5 days old: individual chicks received playbacks in a separate test arena. Playback to chicks was from a loudspeaker [1] with a feeder [2] separated and visually obscured from the released chick by an opaque barrier [4]. The test subject [7] was considered not to have left the release area while it remained within 20 cm of where it had been released into the test arena and was considered in the loudspeaker zone when it was within 20 cm of the loudspeaker [3]. Note that the release area was a circle with 20 cm diameter, while the loudspeaker zone was a half-circle with 20 cm diameter (i.e. half the size of the release area). The arena was filmed using a Raspberry Pi with a camera module [6], and sound recorded using a microphone [5] (we placed a second dummy microphone on the opposite side of the arena).

We conducted this work at the University of Exeter, Streatham Campus between June and November 2021. Experimental manipulations and data collection consisted of different parts, as outlined in figure 1.

#### 2.1.1. Incubation playback (independent samples)

Half the eggs in each of the two cohorts received playbacks of maternal cluck calls during the last week of incubation (incubation-call treatment), while the other half did not receive a playback (incubation-control treatment); see figure 1*a*.

#### 2.1.2. Rearing

Once hatched, chicks were separated into groups of six individuals and reared until they were 21 days old. All chicks within a group had the same incubation treatment, i.e. groups with ‘incubation-call treatment’ and groups with ‘incubation-control treatment’.

#### 2.1.3. Playback during early stages of rearing (repeated measures)

When chicks reached 3−5 days old, they each received three 5 min playback treatments in a random order on consecutive days, while isolated in an arena: (i) control background sound, (ii) chick pleasure-note calls (hereafter ‘chick calls’) or (iii) maternal cluck calls (matching those heard before hatching); see figure 1*c*. Chick pleasure-note calls were chosen as a control as all tested chicks were familiar with these vocalizations at the time of testing and they are emitted in non-threatening contexts. Data were collected on individual vocal and movement behaviour in response to the different stimuli.

#### 2.1.4. Observations during late stages of rearing (repeated measures)

From day 1 onwards, we conducted daily observations of the chick’s behaviour (figure 1*b*) within their home enclosures. First, we recorded a 1 h video each morning from 06.00 to 07.00 and, second, we took high-resolution images at 5 min intervals between 17.00 and 20.00. From the latter, we calculated a social network for each group for the first 14 days of rearing. From the videos, we observed individual perching times and durations as well as visits to other group enclosures.

### 2.2. Chick rearing

We reared two cohorts of chicks from egg to approximately three weeks of age. The first cohort was a common layer hybrid and, due to supplier issues, the second cohort was Hubbard JA 87. See electronic supplementary material, table S1, for details on incubation time and the number of eggs incubated in each cohort. We incubated each cohort of eggs using four Brinsea 56 advanced incubators. During incubation, the temperature was kept at 37.5°C and humidity at 45% in the first two weeks; we turned on periodic cooling between day 7 and day 18 of incubation for 30 min. Eggs were exposed to low levels of indirect light through a small window in the room with the incubators for 10–13 h per day. Once the eggs were set for hatching (on day 18 of incubation), humidity was increased to 70%. Eggs were candled (looking into a developing egg by shining a bright light through it) after 7 and 14 days of incubation. On day 14, we removed any undeveloped eggs from the incubators and the remaining eggs were redistributed evenly across the four incubators.

During the last week of incubation, we played back maternal cluck calls to the eggs in two of the incubators (incubation-call treatment) on average on the hour for about 12 min between 06.00 and 20.00 every day until the end of hatching. The eggs in the remaining two incubators did not experience a playback (incubation-control treatment) and were acoustically isolated from the playbacks. For playbacks, we placed small USB loudspeakers (Honk, Model HK-5002) within the incubators; loudspeakers were connected to a Raspberry Pi 3 Module B+, which remained outside the incubator. See electronic supplementary material, table S1 for details on the dates of incubation playbacks.

Once all the chicks in a cohort had hatched, we turned the playback off and removed the chicks from the incubators. Thus, some of the earlier hatching chicks within the incubation-call treatment incubators were exposed to the maternal cluck calls for a short amount of time after hatching. Chicks were then individually marked with plastic leg rings as well as coloured dots on their heads, lower back and, in some instances, the shoulders with Bic permanent markers. In cohort 1, we sexed the birds by down colour. In cohort 2, we did not manage to precisely feather sex the chicks, meaning that sex was unknown for these birds. We then divided chicks from each cohort into three groups of six birds from the incubation-call treatment and three groups from the incubation-control treatment (*n* = 36 chicks per cohort). Superfluous chicks were either rehomed within 4 days of hatching (*n* = 19) or euthanized using a schedule 1 method (*n* = 3). Chicks were housed in six replicated enclosures (floor space: 180 cm × 122 cm, walls 60 cm height) within the same room, floored with cardboard chippings and containing an adjustable heat lamp, feeder, grit and water, two wooden barriers (50 cm height) and perches (10 and 20 cm height; figure 1). We added vitamins (Nettex Vit Boost) to the water at the very start of rearing, but not thereafter. The room temperature was kept at approximately 25°C and the humidity at 40%. Due to space constraints, we could not keep the chick enclosures acoustically separated from each other. Birds were checked daily to ensure overall health and well-being. Once a week, we exchanged each individual’s rings for larger ones and weighed and measured each bird’s right wing and tarsus. We conducted a *post hoc* analysis of these growth data, which can be found in detail in the electronic supplementary material. Overall, we found that in cohort 1, the laying hen line, incubation-call chicks gained weight slower than control chicks and incubation-call males had a shorter tarsus than control chicks (electronic supplementary material, figure S1). In cohort 2, the broiler line, where we were not able to determine chick sex, we found no such effects, indicating some differences in growth between the two lines (electronic supplementary material, figure S2).

We started the playback trials (see §2.4.1) when chicks were 3 days old, conducting one trial per day per chick. When chicks reached about three weeks of age, they were checked by a vet and rehomed. In cohort 1, we reared 9 females and 9 males with the incubation-control treatment and 10 females and 8 males with the incubation-call treatment. While our incubation treatment might affect the development of males and females differently, we did not include bird sex in any of our analyses presented in the main text (though see the analysis of individual growth in the electronic supplementary material), as it was only known for one cohort and including the variable would have led to overfitting of the models.

### 2.3. Sound recordings for playbacks

We created the incubation-call playbacks from recordings of maternal cluck calls emitted by hens while brooding their eggs from two sources: (i) those provided by ‘The English Country Life’ and (ii) our own recordings of a hen in Griesbach, Germany (see electronic supplementary material, Methods, for details on hen call recordings). These recordings were used in both the incubation-call treatment and the post-hatching playback tests. We added maternal calls from Field *et al.* [30], provided by S. Toukhsati, to expand our set used in the playback test. See electronic supplementary material, tables S2 and S3, for details on the number of hens and calls used for the generation of our playback tracks. We created the background soundtracks for the post-hatching playback tests from recordings of three 5 min segments of sound from the laboratory next to the room containing the chick enclosures. This was composed mostly of low frequencies produced by the ventilation system and was chosen because chicks were familiar with it from incubation and housing in a laboratory environment. We created the chick-call tracks for the post-hatching playback tests from recordings of a separate cohort of layer hen chicks, made for 5 min when they were 3 days old using an Eagle omnidirectional condenser microphone (600 Ω) connected to a laptop computer with the program Audacity 2.4.2. As all chicks were housed in one room, we recorded only one file. From this sound file, we extracted 112 separate calls (i.e. calls where no call was overlayed by any other call).

### 2.4. Playback generation

Each sound file was processed in Adobe Audition (2021). First, we selected calls with minimal background sound (such as traffic, bird song and conspecific calls) and then applied the noise-reduction process in Audition. We then matched the amplitude across sound files within each call type. For the incubation-call treatment, the target amplitude was 16 LUFS. For the cluck-call and chick-call treatments in the post-hatching tests, the target amplitude was 20 LUFS. We proceeded in a similar way with the background sound; background-sound segments extracted from the original sound files ranged from 0.06 to 0.45 s (mean ± s.d. = 0.18 ± 0.07 s) and the target amplitude was 20 LUFS.

We generated playback files in R (R v. 4.0.3; R Core Team, 2020) using the packages seewave [31] and tuneR [32]. As we wanted to distribute the calls in our playbacks following a natural temporal pattern, we first calculated the natural time difference between marked calls of each call type (see electronic supplementary material, table S2, for details on the distribution of times between consecutive calls). We then separated each call by a period of silence, the duration of which was drawn from the previously calculated time-difference distributions (see electronic supplementary material, Methods, for a more detailed description).

For the incubation-call treatment, we generated three files (mean ± s.d. duration = 13.4 ± 0.9 min, range = 12.5 to 14.1 min). We appended the three files to each other, with 45 min silence added in between the call periods, to create a single file of 2 h and 13 min duration. We then reduced the volume to −33 to −27 dB using Adobe Audition 2021 as incubation calls are not naturally very loud. For the post-hatching playback tests, we generated three different files for each of the three treatments (cluck call, chick call and control), each of 5 min. Chicks with incubation-call treatment were probably familiar with some of the calls of the cluck-call treatment, as some of the calls were recorded from the same hens as for the incubation-call treatment (see also electronic supplementary material, tables S2 and S3).

#### 2.4.1. Playback experiment with chicks

To test how individual chicks aged 3−5 days old responded to different sounds depending on their pre-hatching experience of calls, we used an arena (194 cm × 94 cm; walls of 60 cm height) with a centrally placed opaque barrier (approx. 60 cm × 60 cm). On one side of the barrier, we placed a loudspeaker and a feeder. On the other side of the barrier, we released one chick (figure 1*c*). Chicks were released into a dark, turned-over flowerpot (40 cm diameter) with a hole in the bottom by which the chick could be placed into the release area underneath the pot. At the start of the trial, the flowerpot was pulled up and out of sight, with the playback track starting simultaneously. During each trial, we played back 5 min of one of the three possible treatments (chick treatments): (i) maternal cluck calls, (ii) chick calls or (iii) background sound using a small USB loudspeaker (Honk, Model HK-5002) connected to a Raspberry Pi 3 module B. Each trial was recorded using a camera module v. 2 and a USB microphone (GOBEST), both of which were suspended above the test arena and connected to a Raspberry Pi. Each chick was tested on three consecutive days, receiving one treatment per day, in a counterbalanced order such that each of the six chicks in a housing group experienced one of the six possible orders of the three treatments. On each test day, all chicks of one group were tested, before testing the chicks of the next group to reduce overall disturbance associated with catching the birds. We tested 18 chicks in the morning and 18 chicks in the afternoon, rotating the arena after testing nine chicks (we counterbalanced the rotation between test days). Chicks that were tested in the morning on one day were tested in the afternoon the next and vice versa. This resulted in a total of 108 trials per cohort.

From the video and audio recordings of each trial, we recorded the latency of the chick to go around the barrier to the other side(s); the latency to enter the loudspeaker zone(s); the duration spent within the loudspeaker zone(s); and the type (distress calls or pleasure-note calls), number, timing and duration of vocalizations emitted by the chick. Calls in recordings were automatically detected using an amplitude thresholding approach in R using the libraries tuneR [32] and seewave [31]. We checked each recording in Adobe Audition 2021 and manually labelled any missing calls or corrected mislabelled calls (e.g. labelled playback calls).

All statistical analyses were conducted in R 4.0.3 [33]. We used mixed-effects models to account for repeated testing of the same individuals. For each of the analyses, we tested that the model assumptions were met using DHARMa [34]. We used the ‘piah’ package in R [35] for *post hoc* analysis and calculation of *p* values from the mixed-effects models. Eight of the 72 tested chicks were mistakenly given the same playback treatment twice, so were not included in any analyses; this resulted in a sample size of 192 trials on 64 individuals unless otherwise stated.

Chicks did not move away from the release area in 65 out of 192 trials, hence we first tested whether there was any movement around the barrier and towards the loudspeaker zone by fitting a generalized linear mixed model (GLMM) with a binomial distribution (outcome: movement around the barrier, no movement around the barrier). We included the day of testing, incubation treatment, chick treatment and the interaction between incubation treatment and chick treatment as fixed effects, and chick identity nested in cohort identity as random effects. For those chicks that did engage in any exploration (*n* = 127 trials on 58 individuals), we investigated factors affecting the latency to arrive at the loudspeaker zone and factors affecting the time spent within one body length of the loudspeaker zone, using a GLMM with a negative binomial distribution for each analysis. We did not analyse the latency to cross the barrier as it was strongly correlated with the latency to arrive at the loudspeaker zone (Kendall rank correlation test: *τ* = 0.726, *z* = 13.776, *p *< 0.001). For the models examining latency to arrive at the loudspeaker zone and time spent within one body length of the loudspeaker zone, we included incubation treatment, chick treatment and their interaction as fixed effects, and accounted for chick identity and cohort by including them as a nested random effect.

We investigated how incubation treatment and chick treatment affected the latency to emit any vocalizations since the start of the playback trial, with two separate Kruskal–Wallis tests, correcting for multiple testing using a Bonferroni correction; the distribution of the residuals precluded fitting of mixed models. As we found a significant overall effect of chick treatment, we used pairwise Wilcoxon tests to investigate how each chick treatment affected the latency to respond vocally. We then investigated factors affecting the number of distress calls emitted by fitting an LMM. We included the day of testing, incubation treatment, chick treatment and the interaction between incubation treatment and chick treatment as fixed effects, and individual identity nested in cohort identity as random effects. Because chicks emitted far fewer pleasure-note calls than distress calls during our trials, we could not test whether there was a difference in the number of these vocalizations per treatment. Instead, we investigated factors affecting the likelihood of chicks to emit pleasure-note calls (i.e. emission of pleasure-note calls treated as a binary outcome). We fitted a GLMM with a binomial distribution, including chick treatment, incubation treatment and the day of testing as a predictor variable, and individual identity nested in cohort identity as random terms. Due to further convergence problems, we used the ‘bobyqa’ optimizer instead of the default lme4 optimizer [36].

### 2.5. Social and exploratory behaviour during the later stages of rearing

We set two Raspberry Pi cameras (module V2) 1.6 m above each enclosure, connected to a Raspberry Pi 3B+ on a wooden beam, such that they covered most of the enclosure area. Each Pi was programmed to record a 1 h video each morning from 06.00 to 07.00 and one high-resolution image at 5 min intervals between 17.00 and 20.00. On some days, the camera modules got knocked out of place during husbandry checks, meaning that on those days (until the change in position was detected) not all parts of an enclosure were visible, these times were excluded for the calculation of social networks.

#### 2.5.1. Contact between chicks within enclosures

From the time-lapse images, we generated weighted proximity networks where each node represented an individual and each edge represented interactions between individuals. Due to interference of husbandry care, we omitted the first hour of time-lapse data from further analysis. This left 24 images per group per day. We considered individuals to be in proximity when they were located within one body length of one another. To avoid potential bias, data processing was conducted by a student who was blind to incubation treatment. We used R v. 4.0.3 [33] and igraph [37] for further data processing and analysis. Specifically, we generated weighted proximity networks for each group using the association data of the first 14 days of chick rearing (see electronic supplementary material for an analysis of daily proximity networks). In each network, individuals were represented as nodes and the edges represented the normalized number of times individuals were within one body length of each other, calculated as the number of interactions divided by the total number of instances when all chicks were visible in the images. We normalized the weighted degrees to allow us to compare networks across groups (i.e. accounting for different numbers of missing images per group due to knocked cameras or additional husbandry activities). Hence, we only used images when we could account for all individuals per group. Due to technological problems, we only had data for a total of 10 groups (six groups with pre-hatching playback and four without pre-hatching playback, *n* = 60 individuals) to calculate social networks. To test whether chicks from groups that had heard maternal cluck calls during incubation interacted overall more than chicks that had not heard a playback during incubation, we fitted an LMM with treatment as the explanatory variable and group as a random term.

#### 2.5.2. Exploratory behaviour between enclosures

Chicks started perching on the wooden barriers within each enclosure at approximately 17 days after hatching. From the daily video recordings, we noted the timing, duration and identity (ID) of chicks perching on these barriers. This provided us with a measure of perching activity for each bird. We also noted when chicks hopped onto the wooden walls separating neighbouring pens and thus out of the Pi camera’s field of view, as well as when chicks were seen in another group’s enclosure. Chicks could either hop directly from the enclosure wall into the neighbouring enclosure or they could walk along this wall onto the tables in the middle of the room and from there to all other enclosures. Whenever a chick visited another group, classed as the chick standing on the floor or perches of an enclosure that was not their own, we noted its ID as well as the start and end time of the visit (if it was within the hour of video observation). This provided us with a measure of each chick’s exploration activity.

To test whether chicks that had heard maternal cluck calls during incubation were more exploratory than chicks which had been incubated without a playback, we fitted four different GLMMs. As we hypothesized that social connectedness within their home enclosure might explain chick perching and exploratory behaviour, we further tested whether individual weighted degree was correlated with visiting behaviour. Specifically, we imagined two possibilities: (i) chicks with high individual weighted degree might be more likely to visit other groups, being overall more social and hence more likely to seek out additional interactions with audible chicks and (ii) less-connected individuals might be more likely to perch and hop into other enclosures to avoid interactions with their own group members. Thus, we included incubation treatment and normalized individual weighted degree as explanatory variables in each of the GLMMs, as well as group identity nested within cohort identity as random terms. In the first model, we tested whether chicks that had been incubated hearing the maternal cluck calls were more likely to perch on the within-enclosure barriers than chicks which had not heard a playback during incubation, fitting a GLMM with a binomial distribution. In the second model, we fitted a GLMM controlling for zero inflation and with a negative binomial distribution, to examine whether chicks with incubation playback perched for longer on within-enclosure barriers than chicks with no incubation playback. Next, we fitted a binomial GLMM to examine whether chicks with incubation playback were more likely to hop onto the enclosure walls and out of sight than chicks incubated without a playback. Finally, to test whether chicks that had heard playbacks before hatching were more likely to visit other groups, we scored the number of times that chicks were observed in an enclosure that was not their own and fitted another GLMM controlling for zero inflation, with a Poisson distribution.

## 3. Results

### 3.1 Movement response at 3–5 days to playbacks of maternal calls, chick calls and background sound

**Figure 2.**
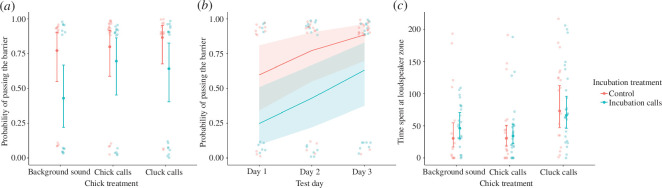
Chick movement during playback trials: the probability that a chick moved away from the release area and around the barrier within a 5 min trial as a function of (*a*) incubation treatment and (*b*) day of testing (trials per chick treatment: *N*_incubation calls_ = 30, *N*_control_ = 34), and (*c*) time spent at the loudspeaker zone. Translucent circles show raw data from individual chicks. Solid lines show estimated model regression, and shaded areas indicate 95% confidence intervals on the fitted regression line. Opaque-filled circles show model estimates and bars show confidence intervals calculated from GLMMs.

During the postnatal playback trials, chicks in the incubation-call treatment were less likely to explore the arena and move around the barrier (47 out of 90 trials) compared with chicks with no pre-hatching sound stimulation (33 out of 99 trials) (GLMM: χ^2^ = 4.67, d.f. = 1, *p* = 0.031; figure 2*a*). This likelihood of exploring the whole test arena and towards the loudspeaker zone increased on consecutive testing days (χ^2^ = 10.61, d.f. = 1, *p* = 0.001; figure 2*a*), but was not significantly affected by the playback type that chicks experienced in the postnatal trial (chick treatment: χ^2^ = 2.94, d.f. = 2, *p* = 0.230). Similarly, there was no significant interaction between chick treatment and incubation treatment (χ^2^ = 1.17, d.f. = 2, *p* = 0.558). In addition, the day of testing (χ^2^ = 0.45, d.f. = 1, *p* = 0.501), incubation treatment (χ^2^ = 0.07, d.f. = 1, *p* = 0.790), chick treatment (χ^2^ = 4.46, d.f. = 2, *p* = 0.107) and their interaction (χ^2^ = 1.257, d.f. = 2, *p* = 0.534) did not significantly affect the arrival time of chicks in the loudspeaker zone.

Chick treatment (GLMM: χ^2^ = 20.96, d.f. = 2, *p* < 0.001; figure 2*b*), but not the day of testing (χ^2^ = 0.68, d.f. = 1, *p* = 0.409), incubation treatment (χ^2^ = 0.15, d.f. = 1, *p* = 0.701) or the interaction between incubation treatment and chick treatment (χ^2^ = 1.89, d.f. = 2, *p* = 0.388), had a significant effect on the time that chicks spent at the loudspeaker zone. Specifically, chicks spent more time within the loudspeaker zone during cluck-call playbacks than during chick-call playbacks (*p* = 0.002) or background-sound playbacks (*p* = 0.008). This comparison was only done for chicks that went around the barrier between the release area and the loudspeaker zone.

### 3.2. Vocal response at 3–5 days to playbacks of maternal calls, chick calls and background sound

**Figure 3.**
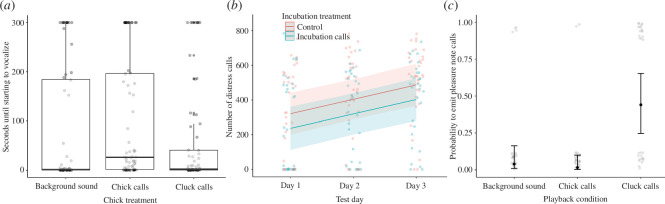
Chick vocalizations during playback trials. (*a*) Latency to vocalize since the start of trial as a function of chick treatment (*x*-axis); points at *y* = 300 indicate chicks that did not vocalize at all. (*b*) Number of distress calls as a function of the day of testing and incubation treatment. (*c*) Probability of emitting pleasure-note calls as a function of chick treatment. Transparent points show raw data, solid points show estimated model coefficients and whiskers indicate 95% confidence intervals on fitted coefficients (see the text for model details). Similarly, solid lines show estimated model regression and shaded areas indicate 95% confidence intervals on the fitted regression line.

Chicks emitted mostly distress calls during the experiment (97.5% of vocalizations) with some pleasure-note calls (2.5% of vocalizations). Chicks that had been incubated in the incubation-call treatment did not differ significantly from the incubation-control chicks in their latency to start vocalizing during playback experiments (chicks with pre-hatching playback: median = 2 s, range = 0−300 s; chicks without pre-hatching playback: median = 1 s, range = 0−300 s; Kruskal–Wallis test: χ^2^ = 2.30, d.f. = 1, *p* = 0.259). However, there was a significant effect of chick treatment (χ^2^ = 10.07, d.f. = 2, *p* = 0.013; figure 3*a*), with chicks starting to vocalize later in response to chick calls (median = 26 s, range = 0−300) compared with background sound (median = 1 s, range = 0−300; paired Wilcoxon test: *p* = 0.010) and cluck calls (median = 2 s, range = 0−300; *p* = 0.010). There was no significant difference between the latency to respond to background sound or cluck calls (*p* = 0.816).

The number of distress calls emitted by chicks during the playback trials was significantly affected by the testing day (LMM: χ^2^ = 21.70, d.f. = 1, *p* < 0.001; figure 3*b*), with chicks increasing their call rate on consecutive days. There was also a non-significant correlation between distress call rate and incubation treatment (χ^2^ = 3.45, d.f. = 1, *p* = 0.063), with control chicks emitting slightly more calls than chicks with incubation-call treatment (figure 3*b*). Chick treatment (χ^2^ = 1.04, d.f. = 2, *p* = 0.594) and the interaction between incubation treatment and chick treatment (χ^2^ = 0.38, d.f. = 2, *p* = 0.827) did not significantly affect the number of distress calls.

In contrast, chick treatment significantly affected the likelihood that pleasure-note calls were emitted (GLMM: χ^2^ = 21.13, d.f. = 2, *p* < 0.001; figure 3*c*). These calls were mostly elicited during cluck-call trials rather than during background-sound trials (*p* < 0.001) or chick-call trials (*p* < 0.001). Incubation treatment (χ^2^ = 0.001, d.f. = 1, *p* = 0.974) and the day of testing (χ^2^ = 2.28, d.f. = 1, *p* = 0.131) did not significantly affect the likelihood that chicks emitted pleasure-note calls.

### 3.3. Differences in social interactions and activity within enclosures in response to pre-hatching playbacks of maternal calls

**Figure 4.**
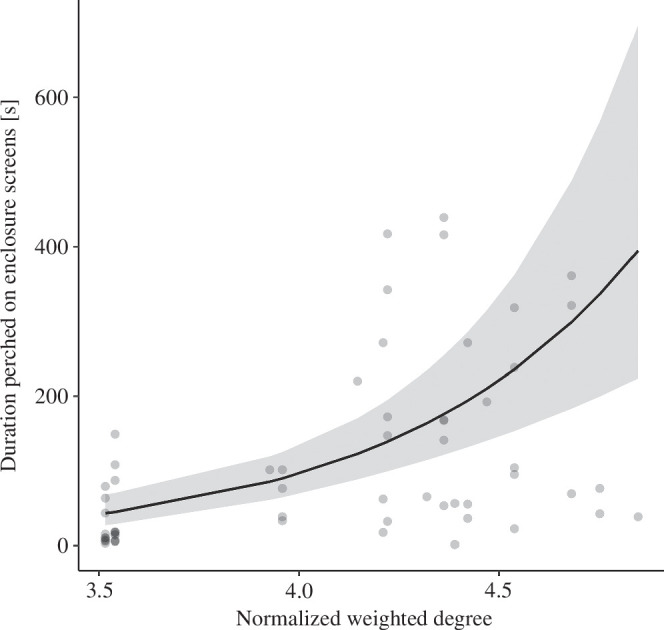
Model predictions from GLMMs showing the perching duration on within-enclosure barriers is dependent on individual weighted degree; *n* = 60, grey circles show raw data from individual chicks. Solid lines show estimated model regression, and shaded areas indicate 95% confidence intervals on the fitted regression line from the GLMM.

**Figure 5.**
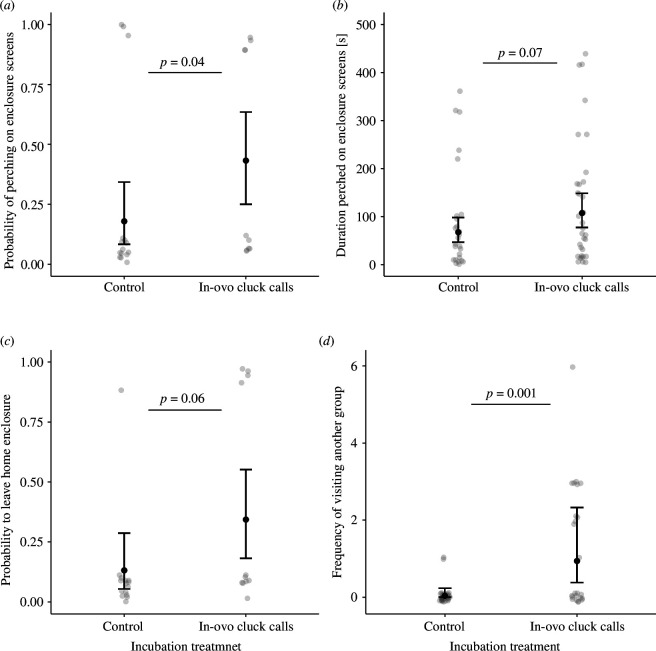
Effect of incubation treatment on perching and ‘visiting’ behaviour. (*a*) The probability to hop on within-enclosure barriers is dependent on incubation treatment. (*b*) The perching duration on within-enclosure barriers is independent of incubation treatment. (*c*) The probability to hop onto enclosure walls is dependent on the incubation experience. (*d*) The frequency of visiting another group by entering that group’s enclosure is dependent on incubation experience. Number of chicks studied: *N*_incubation calls_ = 36, *N*_control_ = 36, grey circles show raw data from individual chicks. Black circles show predicted model estimates and bars show confidence intervals calculated from the GLMMs.

Groups comprising incubation-call chicks did not differ significantly from those comprising incubation-control chicks in the amount of time spent within close proximity to conspecifics within the first two weeks after hatching (normalized weighted degree, LMM: χ^2^ = 0.26, d.f. = 1, *p* = 0.62). There was also no significant difference in the probability that chicks perched on within-enclosure barriers depending on their social connectedness (GLMM: χ^2^ = 1.60, d.f. = 1, *p* = 0.21). Chicks could perch and be in close proximity to each other, hence these two behaviours were not inherently mutually exclusive. However, chicks that overall spent more time in close proximity to conspecifics (high weighted degree) perched on for significantly longer than chicks that overall spent less time in close proximity to group members (χ^2^ = 29.90, d.f. = 1, *p *< 0.001; figure 4). Furthermore, chicks that had experienced the incubation-call treatment were slightly more likely than those from the incubation-control treatment to perch on within-enclosure barriers (χ^2^ = 4.06, d.f. = 1, *p* = 0.04; figure 5*a*). We also found a non-significant trend for incubation-call birds to spend slightly more time perching on within-enclosure barriers than incubation-control birds (χ^2^ = 3.35, d.f. = 1, *p* = 0.07; figure 5*b*).

There was a nonsignificant trend for chicks in the incubation-call treatment to be more likely to hop onto enclosure walls than those from the incubation-control treatment to hop onto enclosure walls (GLMM: χ^2^ = 3.42, d.f. = 1, *p* = 0.06; figure 5*c*). Chicks that had experienced the incubation-call treatment entered another group’s enclosure on average three times more frequently than chicks that did not hear a playback during incubation (χ^2^ = 10.48, d.f. = 1, *p* < 0.001; figure 5*d*). More specifically, 16 out of 36 chicks with pre-hatching playback visited another group on average three times (range 1−6 times) while only three out of 36 chicks without pre-hatching playback visited another group, and did so only once on average within the 1 h observation period. This pattern of leaving or visiting other enclosures was not predicted by measures of sociality within an enclosure, with no significant effect of individual normalized weighted degree on the probability to perch on enclosure walls (χ^2^ = 0.69, d.f. = 1, *p*= 0.41) or the probability to visit another group (χ^2^ = 2.04, d.f. = 1, *p* = 0.15).

## 4. Discussion

Our findings indicate that exposure to maternal cluck calls during the last week prior to hatching affected some variables, but not others, for traits related to movement, vocal behaviour and growth during the first three weeks after hatching. Incubation-call chicks were less likely to explore a novel arena at 3−5 days old but appeared overall slightly more prone to perch on within-enclosure barriers and enclosure walls. This increased exploration of the environment may explain why, by around 17 days old, the incubation-call chicks were more than three times more likely to visit another enclosure than incubation-control chicks. Incubation-call chicks tended to emit fewer distress calls than incubation-control chicks and at least in the laying line grew slower than control chicks. Our findings are broadly consistent with previous work demonstrating how *in ovo* soundscapes alter post-hatching behaviour of domestic chicks [27,38] and other species [7,18,39] and highlight that the impact of such experiences may change with context.

Our data provide insights into how exposure to maternal calls before hatching affects post-hatching behaviour. We believe that the chicks treated the playbacks at age 3−5 days as biologically relevant because, regardless of incubation treatment, they spent more time within the loudspeaker zone when the loudspeaker was playing maternal calls than when it played chick calls or background sound. This was the case even though all chicks had experienced their own and the vocalizations of 35 other chicks (chick calls) throughout the 2 days prior to the first postnatal trial. Thus, if chicks were simply responding to calls with which they had prior experience, we would expect as strong a response to chick calls as to maternal calls.

In our study, all chicks took longer to start vocalizing when they could hear the calls of other chicks compared with maternal calls or background sound. One possible explanation for this difference might be the habituation of our chicks to ‘chick calls’. Our chicks were housed in a single room with little sound insulation between the different enclosures for two days after hatching and before the start of the experimental trials. Hence, chicks had pre-exposure to conspecific sound from visible (own group) and invisible sources (i.e. from neighbouring groups) and this repeated exposure over time may have made them less responsive to chick calls during the playback trials. In contrast, we do not believe that chicks had the same opportunity to habituate to the background sound played during the playback experiment trials, even though the original sound was recorded within the same laboratory environment (the ventilation system). This is because the background sound in the trials was non-continuous and interrupted to mimic chick-calling behaviour. An alternative explanation is that because chicks were used to the acoustic and physical presence of other chicks (in the two days prior to the experiment), they anticipated other chicks being physically present. Thus, they would be less likely to start distress calling, starting only once they realized that no other birds were actually present. Similarly, the absence of chick calls would make them more likely to produce distress calls, regardless of other sounds being played.

Our results suggest that pre-hatching exposure to maternal cluck calls altered the propensity to move and perhaps explore the environment when young. Soon after hatching, and in a novel and likely stressful environment (isolated in the test arena), incubation-call birds were less likely to move and explore, even when calls that might be attractive or even comforting (maternal clucks) could be heard. Chicks, therefore, might be showing higher emotional reactivity and experience our open-field setup as a high-risk environment, where the safest option is to freeze and thus not draw attention from potential predators. This is in line with previous work, suggesting that prenatal acoustic stimulation prompts the general maturation of the auditory system and normal social responses, such that post-hatching chicks respond more accurately to different call types and contexts after hatching [18–21]. By moving away from the release area, chicks might instead try to reinstate social contact with conspecifics. This is also in line with our result, that chicks were more likely to explore the test arena on consecutive testing days, compared with the first day, when the test arena was a totally new environment.

Chicks were also more likely to emit distress calls on consecutive testing days. While distress calls have been shown to be a good indicator of individual stress in domestic chicken chicks with those giving fewer such calls being considered to be less stressed [28,29], they also are an appropriate response by chicks that have become lost, and try to get into contact with their mother. Thus, higher distress call rates on consecutive testing days indicate that the chicks were either more stressed and/or more inclined to reinstate social contact with their conspecifics, which they had spent more time with, in the interim period. In our study, we found no significant difference in the distress-call rate between incubation-call and incubation-control chicks, indicating no difference in the social motivation of chicks depending on incubation treatment. By approximately 14 days, incubation-call birds spent slightly more time exploring their home environment and perching on raised barriers within their home enclosures. In addition, a few days later they were significantly more likely to leave their home environment and enter novel enclosures than incubation-control birds. In these later-life conditions, there was no exposure to maternal calls that could have acted as attractants, so we conclude that the pre-hatching exposure altered more general behavioural activity, specifically exploratory propensity, rather than priming the birds to seek out or respond to those specific calls.

Exposure to maternal calls before hatching does not appear to prime chicks to be inherently more or less likely to generally seek out social partners. During the arena test at 3−5 days old, incubation-call chicks were less likely than incubation-control chicks to approach the loudspeaker at all, but this was regardless of whether it was playing conspecific calls or background noise, so we cannot conclude that the difference was motivated by differences in social affinity. Between 1 and 14 days, within enclosures and before chicks started to move between them, incubation-call and incubation-control chicks did not differ in the amount of close contact with other group members. This suggests that when offered the same opportunities to associate with enclosure mates, the pre-hatching sound environment did not alter the likelihood of chicks to maintain close proximity to conspecifics. However, we did not analyse differences in specific affiliative or agonistic interaction between incubation treatments and it is possible that such differences may exist.

Our work provides further evidence for the effects of prenatal sound exposure, and the lack thereof, on the postnatal behaviour of birds [4,40]. At least in some breeds of domestic chicken, prenatal exposure to maternal calls can affect movement, exploratory behaviour and individual growth after hatching. We cannot determine whether the absence of an effect of prenatal acoustic experience on growth in the second cohort was because of the different breed, or because the effect of sex could not be included due to a lack of phenotypic sex markers. Given that sex interacted with incubation treatment for some morphological traits in the first cohort, combining the data together might lead to the effect being masked. Thus, effects on growth may or may not be present, and potentially differ between both sexes and breeds. Our study shows that chicks respond according to context and age, with incubation-call chicks showing less exploratory behaviour when on their own in an open-field environment at age 3–5 days, and more exploratory behaviour at 17–21 days when in a safe environment surrounded by conspecifics, than chicks with no sound stimulation before hatching. This means that such post-hatching effects of pre-hatching acoustic experiences may be more nuanced and context-dependent (and/or age-dependent) than previously understood. Importantly, in our study, the incubation-control condition (no playback and hence only incubator noise) is more abnormal from an evolutionary point of view, as in the wild, chicks would naturally be incubated by their mother, thus being exposed to her vocalizations and no incubator noise. Rather than the incubation-treatment chicks being stimulated, the control chicks were deprived of stimulation, which is expected to cause developmental effects [41].

While our work contributes to a growing body of evidence that early-life sound exposure can affect later behaviour over the relatively short term [7,15,21,39], we still know little about the ultimate function and longer term consequences of early-life sound exposure (but see [14]). The acquisition of information from the environment by developing embryos is probably widespread in the animal kingdom, with embryos probably using a range of sensory modalities to gain information about their current or future environments. Thus, we recommend further research into the effects of early-life soundscapes on later-life behaviour across multiple contexts, considering both short- and long-term consequences.

## Data Availability

Data and code are available on Dryad [42]. Supplementary material is available online [43].

## References

[1] Groothuis TGG , Hsu BY , Kumar N , Tschirren B . 2019 Revisiting mechanisms and functions of prenatal hormone-mediated maternal effects using avian species as a model. Phil. Trans. R. Soc. B **374** , 20180115. (10.1098/rstb.2018.0115) 30966885 PMC6460091

[2] Lupien SJ , McEwen BS , Gunnar MR , Heim C . 2009 Effects of stress throughout the lifespan on the brain, behaviour and cognition. Nat. Rev. Neurosci. **10** , 434–445. (10.1038/nrn2639) 19401723

[3] Monaghan P . 2008 Early growth conditions, phenotypic development and environmental change. Phil. Trans. R. Soc. B **363** , 1635–1645. (10.1098/rstb.2007.0011) 18048301 PMC2606729

[4] Mariette MM , Clayton DF , Buchanan KL . 2021 Acoustic developmental programming: a mechanistic and evolutionary framework. Trends Ecol. Evol. **36** , 722–736. (10.1016/j.tree.2021.04.007) 34052045

[5] Sheriff MJ , Love OP . 2013 Determining the adaptive potential of maternal stress. Ecol. Lett. **16** , 271–280. (10.1111/ele.12042) 23205937

[6] Hossie T , Landolt K , Murray DL . 2017 Determinants and co‐expression of anti‐predator responses in amphibian tadpoles: a meta‐analysis. Oikos **126** , oik.03305. (10.1111/oik.03305)

[7] Mariette MM , Buchanan KL . 2016 Prenatal acoustic communication programs offspring for high posthatching temperatures in a songbird. Science **353** , 812–814. (10.1126/science.aaf7049) 27540172

[8] Rumpf M , Tzschentke B . 2010 Perinatal acoustic communication in birds: why do birds vocalize in the egg? Open Ornithol. J. **3** , 141–149. (10.2174/1874453201003010141)

[9] d’Ettorre P , Hughes DP (eds). 2008 Sociobiology of communication: an interdisciplinary perspective. Oxford, UK: Oxford University Press. (10.1093/acprof:oso/9780199216840.001.0001)

[10] Espmark Y , Amundsen T , Rosenqvist G (eds). 2000 Animal signals: signalling and signal design in animal communication. Trondheim, Norway: Tapir Academic Press.

[11] Bradbury JW , Vehrencamp SL . 1998 Principles of animal communication. Sunderland, MA: Sinauer Associates.

[12] Lickliter R , Hellewell TB . 1992 Contextual determinants of auditory learning in bobwhite quail embryos and hatchlings. Dev. Psychobiol. **25** , 17–31. (10.1002/dev.420250103) 1740227

[13] Noguera JC , Velando A . 2019 Bird embryos perceive vibratory cues of predation risk from clutch mates. Nat. Ecol. Evol. **3** , 1225–1232. (10.1038/s41559-019-0929-8) 31332329

[14] Katsis AC , Buchanan KL , Kleindorfer S , Mariette MM . 2021 Long-term effects of prenatal sound experience on songbird behavior and their relation to song learning. Behav. Ecol. Sociobiol. **75** . (10.1007/s00265-020-02939-5)

[15] Katsis AC , Davies MH , Buchanan KL , Kleindorfer S , Hauber ME , Mariette MM . 2018 Prenatal exposure to incubation calls affects song learning in the zebra finch. Sci. Rep. **8** , 15232. (10.1038/s41598-018-33301-5) 30323211 PMC6189107

[16] Kleindorfer S , Evans C , Hauber ME , Colombelli-Négrel D . 2018 Could prenatal sound discrimination predict vocal complexity later in life? BMC Zool. **3** , 11. (10.1186/s40850-018-0038-1)

[17] Hudson EJ , Creanza N , Shizuka D . 2020 The role of nestling acoustic experience in song discrimination in a sparrow. Front. Ecol. Evol. **8** , 99. (10.3389/fevo.2020.00099)

[18] Bailey ED . 1983 Influence of incubation calls on post-hatching responses of pheasant chicks. Condor **85** , 43–49. (10.2307/1367884)

[19] Bailey ED , Ralph KM . 1975 The effects of embryonic exposure to pheasant vocalizations in later call identification by chicks. Can. J. Zool. **53** , 1028–1034. (10.1139/z75-118)

[20] Lickliter R , Stoumbos J . 1992 Modification of prenatal auditory experience alters postnatal auditory preferences of bobwhite quail chicks. Q. J. Exp. Psychol. Sect. **44** , 199–214. 10.1080/02724999208250612 1598419

[21] Gottlieb G . 1983 Development of species identification in ducklings: X. Perceptual specificity in the wood duck embryo requires sib stimulation for maintenance. Dev. Psychobiol. **16** , 323–333. (10.1002/dev.420160407) 6884581

[22] Udino E , George JM , McKenzie M , Pessato A , Crino OL , Buchanan KL , Mariette MM . 2021 Prenatal acoustic programming of mitochondrial function for high temperatures in an arid-adapted bird. Proc. R. Soc. B **288** , 20211893. (10.1098/rspb.2021.1893) PMC8651415 34875198

[23] Pessato A , McKechnie AE , Mariette MM . 2022 A prenatal acoustic signal of heat affects thermoregulation capacities at adulthood in an arid-adapted bird. Sci. Rep. **12** , 5842. (10.1038/s41598-022-09761-1) 35393484 PMC8991222

[24] Mezrai N , Houdelier C , Bertin A , Calandreau L , Arnould C , Darmaillacq AS , Dickel L , Lumineau S . 2022 Impact of natural and artificial prenatal stimulation on the behavioural profile of Japanese quail. J. Exp. Biol. **225** , jeb243175. (10.1242/jeb.243175) 35213895

[25] Lickliter R . 2018 The influence of prenatal experience on behavioral and social development: the benefits and limitations of an animal model. Dev. Psychopathol. **30** , 871–880. (10.1017/S0954579418000640) 30068430

[26] Collias NE , Joos M . 1953 The spectrographic analysis of sound signals of the domestic fowl. Behaviour **5** , 175–188. (10.1163/156853953X00104)

[27] Fält B . 1981 Development of responsiveness to the individual maternal ‘clucking’ by domestic chicks (Gallus gallus domesticus). Behav. Processes **6** , 303–317. (10.1016/0376-6357(81)90048-6) 24925863

[28] Manteuffel G , Puppe B , Schön PC . 2004 Vocalization of farm animals as a measure of welfare. Appl. Anim. Behav. Sci. **88** , 163–182. (10.1016/j.applanim.2004.02.012)

[29] Herborn KA , McElligott AG , Mitchell MA , Sandilands V , Bradshaw B , Asher L . 2020 Spectral entropy of early-life distress calls as an iceberg indicator of chicken welfare. J. R. Soc. Interface **17** , 20200086. (10.1098/rsif.2020.0086) 32517633 PMC7328393

[30] Field SE , Rickard NS , Toukhsati SR , Gibbs ME . 2007 Maternal hen calls modulate memory formation in the day-old chick: the role of noradrenaline. Neurobiol. Learn. Memory **88** , 321–330. (10.1016/j.nlm.2007.04.001) 17507256

[31] R Core Team . 2020 R: a language and environment for statistical computing. Vienna, Austria: R Foundation for Statistical Computing.

[32] Sueur J , Aubin T , Simonis C . 2008 Seewave, a free modular tool for sound analysis and synthesis. Bioacoustics **18** , 213–226. (10.1080/09524622.2008.9753600)

[33] Ligges U , Krey S , Mersmann O , Schnackenberg S . 2018 TuneR: analysis of music and speech. See https://CRAN. R-project. org/package= tuneR.

[34] Hartig F . 2020 DHARMa: residual diagnostics for hierarchical (multi-level/mixed) regression models. R package version 0.3.3.0. See https://CRAN.R-project.org/package=DHARMa.

[35] De Rosario-Martinez H . 2015 Phia: post-hoc interaction analysis. R package version 0.2-1.

[36] Bates D , Mächler M , Bolker B , Walker S . 2015 Fitting linear mixed-effects models using lme4. J. Stat. Softw. **67** , 1–48. (10.18637/jss.v067.i01)

[37] Csardi G , Nepusz T . 2006 The igraph software package for complex network research. InterJ. Comp. Syst. **1695** , 1–9.

[38] Jain S , Sharma R , Wadhwa S . 2004 Effect of prenatal species-specific and music stimulation on the postnatal auditory preference of domestic chick. Ind. J. Physiol. Pharmacol. **48** , 174–183. 15521556

[39] Noguera JC , Velando A . 2020 Gull chicks grow faster but lose telomeres when prenatal cues mismatch the real presence of sibling competitors. Proc. R. Soc. B **287** , 20200242. (10.1098/rspb.2020.0242) PMC7287347 32429809

[40] Rivera M , Louder MIM , Kleindorfer S , Liu W , Hauber ME . 2018 Avian prenatal auditory stimulation: progress and perspectives. Behav. Ecol. Sociobiol. **72** , 112. (10.1007/s00265-018-2528-0)

[41] Mariette MM . 2024 Developmental programming by prenatal sounds: insights into possible mechanisms. J. Exp. Biol. **227** , jeb246696. (10.1242/jeb.246696) 38449334

[42] Gall G . Data from: Exposure to calls before hatching affects the post-hatching behaviour of domestic chickens. Dryad Digital Repository. (10.5061/dryad.wm37pvmrw) PMC11321849 39144491

[43] Gall GEC , Letherbarrow M , Strandburg-Peshkin A , Radford AN , Madden JR . 2024. Data from: Exposure to calls before hatching affects the post-hatching behaviour of domestic chickens. Figshare. (10.6084/m9.figshare.c.7389780) PMC11321849 39144491

